# Smisha Agarwal: supporting practical digital innovation

**DOI:** 10.2471/BLT.25.030225

**Published:** 2025-02-01

## Abstract

Smisha Agarwal talks to Gary Humphreys about the challenges faced in introducing digital tools in resource-constrained settings.

*Q: When did you become interested in digital health?*

A: I trained as a dentist, but almost immediately got out of the consulting room to work in the communities I was hoping to help, notably in rural Maharashtra in a community-based organization – the Comprehensive Rural Health Project, Jamkhed – training community health workers and supporting efforts to strengthen primary health-care systems in villages. I was in the process of digitizing community health records for over 100 villages – an incredibly complicated task, in part due to poor infrastructure and power outages – and I started noticing that, despite having lower levels of literacy, most community health workers (CHWs) had cell phones, could keep them charged and were using them to listen to cricket updates, which are data after all. So, I asked myself, “How can we use these phones to capture health data in real time?”

*Q: What was the answer?*

A: The basic idea was to use interactive voice response (IVR), leveraging the mobile phones that CHWs were already using. IVR allowed users to interact with computerized voice commands and numeric keypad inputs to enter pregnancy-related data, which were then uploaded to the cloud in real time. I set up a company in 2008 with a group of friends, and we rolled out an IVR-based scheme in about 40 villages. It was only a small-scale project, but it was very effective, and the CHWs were really motivated by it. Unfortunately, the cost of IVR technology back then was a major limitation, and because of that and other reasons the programme didn’t scale. However, when I arrived in the USA in 2009, I discovered that there was considerable interest in such approaches which were being collectively referred to as mHealth (mobile health). On the basis of the expertise that I had developed with my work in Jamkhed – which included experimenting with different approaches to addressing high-risk pregnancies and creating referral systems – I started supporting the World Health Organization's (WHO) mHealth technical evidence review group. I was then appointed to the faculty at Johns Hopkins, and collaborated with WHO on providing support to a dozen or so digital health programmes globally. Over the past decade, Johns Hopkins and WHO have collaborated on several studies based on direct observations of provider–patient interactions to evaluate how mHealth tools impact providers, support community health workers in taking on higher skilled responsibilities and advance the quality of primary health care. While confirming the beneficial impact of such tools, especially in making data available for decision-making, the research presents a more nuanced picture in regard to health service delivery. We have found, for example, that the impact of mHealth tools is highly dependent on the extent to which they are adapted to local context and use.

“Digitization can often add complexity.”

*Q: Can you say a little more about that?*

A: We have found that tools that are too comprehensive can get in the way of effective service delivery. In the kinds of setting we have been studying, most provider–patient interactions are brief, lasting around five minutes or so. However, the kind of comprehensive protocols that have been developed for decision aids can significantly extend that time. So, while they may ensure thoroughness, they can double or triple the interaction time, not only adding to the CHWs' tasks but making it harder for them to build trust with the patient. The same goes for data capture, which can be very time-consuming. As a result of these and other challenges, we have noticed that health outcomes don’t improve significantly, especially if critical resources – such as diagnostic tests – aren’t available. It’s important to remember that the digital tools are only part of a bigger equation, and that their success depends on how well they are adapted for, and integrated into, the health-care ecosystem in which they are supposed to work.

*Q: To what extent can training help to overcome these challenges?*

A: Training and practice definitely help, but we’ve learned that it is also important to set realistic expectations and to modify the mHealth tools accordingly. If a typical health worker/patient encounter involves five questions, and we suddenly introduce a tool with 20 questions, it quadruples the interaction time. Instead, we focus on identifying what constitutes a “good enough” clinical encounter. For instance, we make certain steps optional to streamline the process and to improve user experience. The proliferation of apps is another issue. Because of the siloed nature of the global funding ecosystem, there are often separate apps for different programmes, which can lead to very cumbersome exchanges between CHWs and patients. I recently observed CHWs in a village in India using separate apps for nutrition, family planning, reproductive care and underweight children, giving rise to some very laborious conversations. The problems are only compounded when CHWs are required to collect the same data again and again to report through different apps.

*Q: What is being done to address these different challenges?*

A: On the data collection front, over the past decade, health ministries worldwide have invested in developing consolidated digital health strategies to scale systems nationally. These typically involve setting up standards for data sharing and interoperability. However, establishing such standards is just the first step. Execution is technically complex and expensive, requiring substantial technical and financial resources.

*Q: To what extent do cultural or regional differences affect digital health adoption?*

A: Our research indicates that cultural and regional differences are important. While cell phones themselves are ubiquitous, the sociocultural contexts in which they are used vary greatly, and this affects how tools are adopted. For instance, in India, some digital interventions deliver direct-to-patient voice messages tailored to guide mothers through their pregnancy. These messages might use the voice of an older woman, creating a relatable persona for the audience. In other regions, a message from the health ministry might carry more authority and be more effective.

*Q: Can you cite examples of programmes where clinical decision-support tools have had a positive impact?*

A: Yes, we’ve seen success in several programmes. For example, in Burkina Faso, a programme implemented by the health ministry in collaboration with partners demonstrated that using clinical decision-support tools improved the quality of care delivered by less-trained workers during prenatal and postnatal visits. Similar community-based programmes in India have shown that these tools help health workers create better referrals and linkages to care for pregnant women. The tools can also expedite data reporting, but not always. For example, in many settings, health workers still do dual data entry – recording data both on paper and digitally. This can slow things down. Additionally, there’s a “big brother” effect, where workers feel they are being monitored in real time. This makes them more cautious, often double-checking their data before uploading them. Technical challenges, such as system glitches or connectivity issues, also play a role.

*Q: How optimistic are you that different challenges you have presented can be overcome?*

A: I’m optimistic, but I also like to think of myself as a pragmatist. There’s a lot of hype around digitization and AI, but technology doesn’t solve everything. In communities with limited infrastructure, education or resources, digitization can often add complexity rather than solving problems. In public health, we must have a “health-first” focused and not "tech-first” focus. To ensure a technology-driven solution impacts lives, we must harmonize technological advances with contextual readiness.

*Q: What do you mean by that?*

A: Simply, that sometimes digitization isn’t the right approach. Digitizing a broken process just creates a broken digitized process. For example, CHWs often collect data on paper, then report it to a supervisor. The broken part of the process is that these workers are often unpaid, unsupervised, and lack incentives and tools to deliver care consistently. If you give them a shiny device to collect data, they might be happy to have it and it might enhance their credibility in the community, but it doesn’t fix the underlying issues of accountability or motivation. And if the device is hard to use, you’ve simply added to their workload. That said, I believe that digital tools are absolutely the future. They are critical for building pandemic resilience and improving health-care delivery in many areas, such as disease surveillance and logistics management. For example, digitizing logistics can improve supply chain efficiency, automate reimbursements, and detect fraud using AI. We’ve already seen clear evidence of impact in specific applications. For instance, text message reminders for immunizations or prenatal and postnatal care improve adherence rates. We’ve also seen some success with chatbots, something I have worked on, particularly in regard to natural language processing (NLP).

*Q: Can you explain what that is?*

A: NLP is a branch of artificial intelligence that enables computers to understand language as text or speech, interpret it, and generate appropriate responses. We have been using NLP to develop chatbots which have immense potential in health care. For example, patients in urban areas may only spend two minutes with a doctor but might spend hours or even days online searching for information about their symptoms. Virtual communities often emerge in these spaces, where people share experiences, misinformation, stigma or shame. Chatbots can help address these issues by providing accurate and timely information in an accessible way, but they also have applications in other areas – counselling, for example, or triage. We’re trying to understand how best they can be developed, applied and scaled. Among the most promising initiatives are those using a “human-in-the-loop” model, where people can interact with a chatbot to initiate services and ask questions but where a human monitors the system and can step in to escalate cases or provide referrals for pregnancy-related services. Several randomized controlled trials are being planned to evaluate the effectiveness of such approaches.

*Q: Despite all the challenges, are you optimistic about the future?*

A: Yes, but we shouldn’t expect overnight results. Digital health is a learning journey. We need to reflect, revise and refine our approaches to make these tools work effectively. My feeling is that the more we temper expectations, the better we can address the real challenges and make meaningful progress.

